# Exploring the Role of Paraoxonases in the Pathogenesis of Coronary Artery Disease: A Systematic Review

**DOI:** 10.3390/ijms151120997

**Published:** 2014-11-14

**Authors:** David Abelló, Elena Sancho, Jordi Camps, Jorge Joven

**Affiliations:** 1 Department of Medicine and Surgery, Faculty of Medicine and Health Sciences, Rovira i Virgili University, C. Sant Llorenç, s/n, Reus, Catalonia 43201, Spain; E-Mails: davidabelloaudi@gmail.com (D.A.); sancho.08@hotmail.com (E.S.); 2 Biomedical Research Unit, Hospital Universitari de Sant Joan, Institut d’Investigació Sanitària Pere Virgili, C. Sant Joan, s/n, Reus, Catalonia 43201, Spain

**Keywords:** atherosclerosis, coronary artery disease, myocardial infarction, oxidative stress, paraoxonase, systematic review

## Abstract

Paraoxonases (PON) are three enzymes (PON1, PON2 and PON3) that play a role in the organism’s antioxidant system; alterations in which are associated with diseases involving oxidative stress. In this review, we summarize the evidence of PON related to the pathogenesis of coronary artery disease (CAD) and atherosclerosis. We searched three electronic databases (PubMed, Scopus and Cochrane Database) with no date limit. All of the articles selected investigated PON enzymatic activity and/or PON gene polymorphisms. The selection focused on PON in relation to atherosclerosis, CAD and myocardial infarction. The exclusion criteria were a sample size <100 patients, non-human studies, editorials and systematic reviews without restrictions on the country of origin. With these criteria, we identified thirty-five prospective studies published between 1986 and 2014 with a total of 28,164 participants. The relationship between PON gene polymorphisms and CAD was not conclusive, but most studies support the concept that alterations in PON1 enzymatic activity levels do influence atheroma formation. Conversely, relationships between PON2 and PON3 *vs.* CAD have not been extensively investigated. Our review of the current data concludes that the bases of paraoxonases involvement in atherosclerosis are poorly understood and that this issue requires future comprehensive, multi-centered studies.

## 1. Introduction

The paraoxonases (PON) are enzymes involved in oxidative stress, in the atherosclerosis process and, consequently, in vascular disease. However, their specific role in these clinical derangements is still under debate, and we sought definitive conclusions by evaluating published studies.

The PON family contains three enzymes: PON1, PON2 and PON3. Their genes are located adjacent to each other on chromosome 7q21-22 [1,2]. Several polymorphisms have been reported for the PON genes in the coding, as well as the promoter regions; alterations that influence the activities and/or concentrations of these enzymes to a greater or lesser extent [2,3,4,5]. PON1 and PON3 are found in many tissues, as well as in circulation, associated with high-density lipoproteins (HDL), while PON2 is exclusively intracellular [6,7,8,9].

PON1 was initially described as an organophosphate hydrolase, and its role in insecticide poisoning has been extensively reported [10]. Subsequent studies demonstrated that PON1 also had a lactonase activity and is able to hydrolyze lipophilic lactones and degrade oxidized lipids in lipoproteins and in cells [11,12]. This property enabled investigators to propose that PON1 plays a key role in the protection against atherosclerosis and its related systemic diseases [1].

PON2 and PON3 are also able to hydrolyze lactones similar to PON1, and they have also been implicated in the atherosclerosis process. Nevertheless, their modes of action have been shown not to be exactly equivalent to that of PON1, and further investigations are needed to fully clarify their roles. The antiatherogenic effect of PON2 could be attributed to the protection of mitochondria against oxidative stress, while PON3 has been shown to be a protective factor against obesity. As such, new and interesting lines of research are opened up. To summarize, the three members of the PON family are related genetically, possess lactonase activity and are involved in the atherosclerosis process [6].

The aim of this systematic review is to update and clarify the scientific evidence available on PON enzymes and their roles in cardiovascular disease. We provide, here, a novel approach integrating *PON* gene polymorphisms, their enzymatic activities and their correlation with atherosclerosis.

## 2. Methodology and Search Strategy

### 2.1. Search Strategy

The search was performed in three electronic databases: PubMed, Scopus and Cochrane. Article selection was performed in July 2014, and no date limit was applied to articles selected. Studies included were restricted to those on humans and to English-language journals. Search terms were combined using Boolean operations. The search terms included: (“Paraoxonases” OR “PON”) AND (“myocardial infarction” OR “CHD” OR “vascular disease” OR “atherosclerosis”). Initially, the referenced articles were manually screened independently by two investigators of the team. The first screening selection was based on the article’s title and a second screening on the abstract. All duplicate publications were omitted.

### 2.2. Selection Criteria

All of the articles involved in PON enzymatic activity and/or PON gene polymorphisms were selected. The selection focused on the PON relationship with atherosclerosis, coronary artery disease (CAD) and myocardial infarction (MI). Other diseases and comorbidities were excluded.

The exclusion criteria were a sample size of <100 patients, non-human studies, editorials and systematic reviews. No country distinctions were applied.

### 2.3. Data Extraction

Data were extracted and pooled and included: year of publication, sample size, type of study, disease observed and the polymorphisms studied. Because serum PON1 possesses a wide catalytic versatility and is able to hydrolyze a variety of substrates [6], the measurement methods of the publications were also considered. Main conclusions were summarized, and missing data were obtained directly from the respective corresponding authors. In some cases, these data were not forthcoming, and the studies were deleted from our analysis.

## 3. Results

### 3.1. Identification of Studies

**Figure 1:**
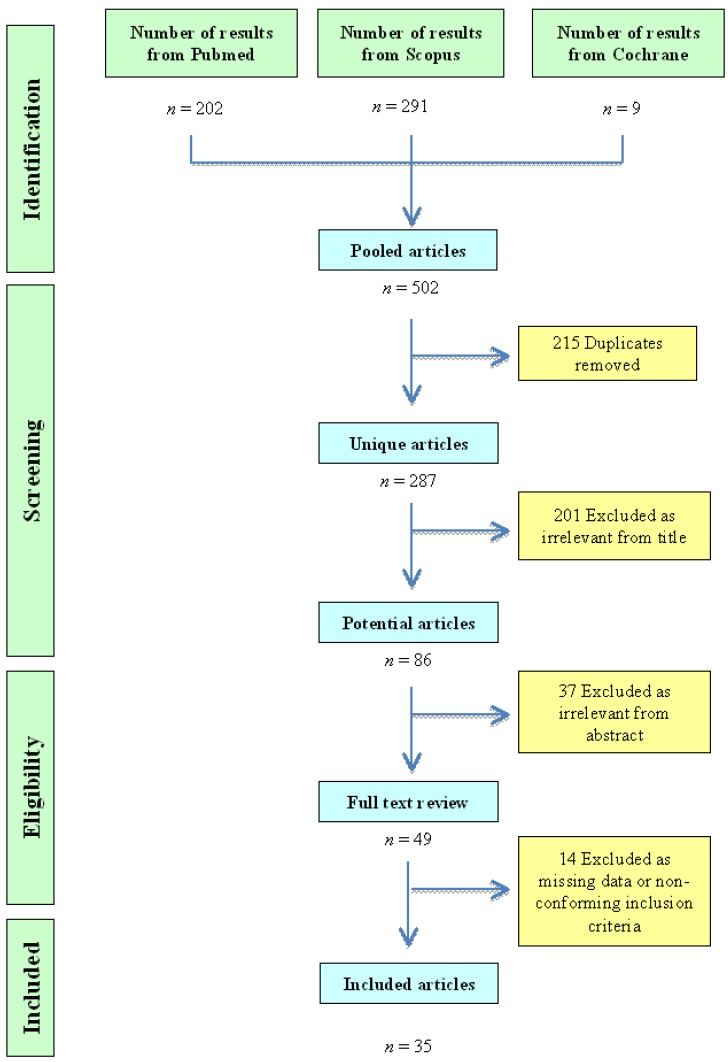
Flow chart of study selection and review.

The initial electronic search of the above-mentioned databases identified 502 articles (Figure 1). After exclusion of duplicates (*n* = 215) and with 201 contributions considered irrelevant based on the titles and 37 on the abstract, the selection was reduced to 49 full-text articles. Subsequently, studies (*n* = 14) with missing data or without compliance with inclusion or exclusion criteria were discarded. Finally, 35 manuscripts were included in this systematic review [13,14,15,16,17,18,19,20,21,22,23,24,25,26,27,28,29,30,31,32,33,34,35,36,37,38,39,40,41,42,43,44,45,46,47]. The process of selection is depicted in Figure 1.

### 3.2. Study Characteristics

The overall study characteristics are displayed in Table 1. All of the articles were prospective studies focusing on the influence of PON enzymes in CAD, MI and atherosclerosis. Most of them were case-control studies. They were published between 1986 and 2014 and included a total of 28,164 participants. Eighteen manuscripts investigated the relationship of the gene polymorphisms with disease, while eight investigated the enzymatic activity in relation to disease; only nine analyzed both variables. The main substrates used to measure PON1 enzyme activity were paraoxon (paraoxonase activity) and phenylacetate (arylesterase activity) in 12 and eight studies, respectively. Some studies employed homocysteine thiolactones as substrates (hcy-thiolactonase activity).

**Table 1:** Characteristics of the included studies. ATH, atherosclerosis; C, cohort; CAD, coronary artery disease; CC, case-control; CS, cross-sectional; EA, enzyme activity; GP, genetic polymorphism; MI, myocardial infarction.

First author	Year	Location of the studied population	Type of study	Patients (*n*)	Disease	Object of study		Enzyme activity measurement	Reference number
						GP	EA		
Liu, T.	2014	China	CC	2456	CAD	√	√	Paraoxonase-arylesterase	13
Shekhanawar, M.	2013	India	CS	110	CAD	-	√	Arylesterase	14
Cozzi, L.	2013	Italy	CC	105	MI	√	-	-	15
Tang, W.H.W.	2012	USA	C	3668	CAD MI	√	√	Paraoxonase-arylesterase	16
Bayrak, A.	2012	Turkey	CC	270	CAD	√	√	Thiolactonase-paraoxonase	17
Saxena, T.	2011	India	CC	150	MI	-	√	Paraoxonase	18
Gluba, A.	2010	Poland	CC	407	MI	√	-	-	19
Lakshmy, R.	2010	India	CC	345	MI	√	√	Paraoxonase	20
Regieli, J.J.	2009	Netherlands	C	793	CAD	√	-	-	21
Jayakumari, N.	2009	India	CC	574	CAD	-	√	Arylesterase	22
Yildiz, A.	2008	Turkey	CC	134	CAD	-	√	Paraoxonase	23
Saeed, M.	2007	Pakistan	CC	581	MI	√	-	-	24
Jarvik, G.P.	2003	USA	CC	437	CAD	√	√	Arylesterase	25
Domagala, T.B.	2006	U.K.	CC	475	CAD	√	√	Thiolactonase-paraoxonase	26
Su, S.	2005	China	CC	423	CAD	√	√	Arylesterase	27
Rodríguez-Esparragón, F.	2005	Spain	CC	619	CAD	√	-	-	28
Kabarolgu, C.	2004	Turkey	CC	103	CAD MI	-	√	Paraoxonase	29
Srinivasan, S.R.	2004	USA	C	1786	ATH	√	-	-	30
Göçmen, A.Y.	2004	Turkey	CC	172	CAD	-	√	Paraoxonase	31
Oliveira, S.A.	2004	Brazil	CC	732	CAD	√	-	-	32
Robertson, K.S.	2003	U.K.	C	3052	CAD	√	-	-	33
Wang, X.	2003	China	CC	949	CAD	√	√	Arylesterase	34
Fortunato, G.	2003	Italy	C	310	ATH	√	-	-	35
Rahmani, M.	2002	Iran	CC	251	CAD	-	√	Paraoxonase-arylesterase	36
Ferré, N.	2002	Spain	CC	215	MI	√	√	Paraoxonase	37
Hong, S.H.	2001	Korea	CC	304	CAD	√	-	-	38
Malin, R.	2001	Finland	CS	123	ATH	√	-	-	39
Gardemann, A.	2000	Germany	C	2784	CAD MI	√	-	-	40
Cascorbi, I.	1999	Germany	CC	2000	CAD	√	-	-	41
Sanghera, D.K.	1998	USA	CC	318	CAD MI	√	-	-	42
Ombres, D.	1998	Italy	CC	472	CAD	√	-	-	43
Sanghera, D.K.	1997	India-China	CC	1034	CAD	√	-	-	44
Suehiro, T.	1996	Japan	CC	386	CAD MI	√	-	-	45
Herrmann, S.M.	1996	France	CC	1343	MI	√	-	-	46
McElveen, J.	1986	U.K.	CC	283	MI	-	√	Paraoxonase	47

### 3.3. PON Genetic Polymorphisms and CAD

We found that the influence of the several PON genetic polymorphisms on CAD was not generally substantiated. Several studies [13,15,19,20,21,24,32,33,34,42,44] found associations between one or more polymorphisms of the PON genes *vs.* the disease. The strongest association was found with PON1 Q192R polymorphism, specifically reporting a protective role of the 192Q allele and a deleterious effect of the 192R allele [13,19,20,24,42,44]. However, conflicting results were reported in some manuscripts [21,34] in which the 192Q allele was more frequent in CAD patients, in contradiction to the other studies. Other PON1 and PON2 polymorphisms (M55L, C-108T, R-160G, G-162A from PON1 and S311C and A148G from PON2) have been associated with cardiovascular disease [24,32,33,34]. One article [32] suggested that the 55M allele of PON1 has a protective effect against CAD, while other studies [21,33] stated the opposite. Some articles [15,33,34,42] found a higher frequency of 311S and 311C alleles in CAD patients. Adding more confusion to the debate, several studies failed to find any kind of relationship between PON polymorphisms and CAD [16,17,25,26,28,35,37,38,39,40,41,43,45,46]. The hypothesis that variations in the PON genes were predictive factors for CAD or MI has also been investigated, and again, the results have been conflicting; some articles [25,32,40,41,43,45] negate this hypothesis, while others [39,40] note a relationship between the 55L and 192R alleles as positively associated with the severity of CAD.

**Figure 2:**
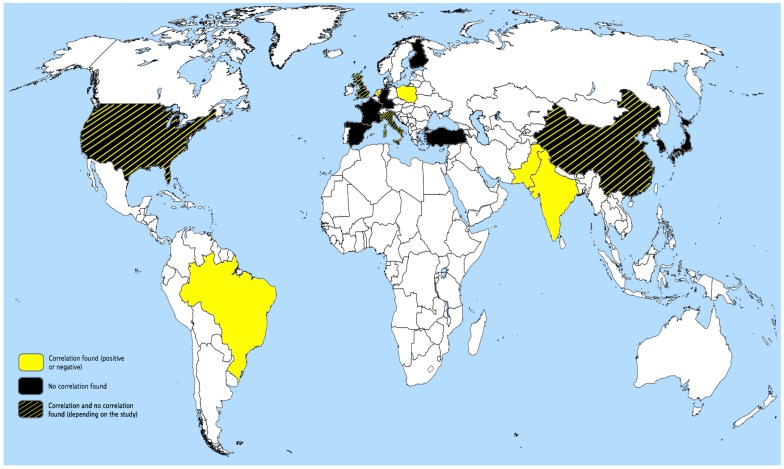
Country of origin and outcomes of studies investigating the possible association between PON gene polymorphisms and CAD.

The possibility remains that these contradictory outcomes result from ethnic differences, as illustrated by comparisons of African Americans *vs.* white Americans [30] or Indian *vs.* Chinese populations [44]. However, these findings appear to be anecdotal, and we could not find any association between ethnicity or country of origin and the influence of PON gene polymorphisms on CAD. Additionally, where countries provide more than one study, the conclusions are contradictory in terms of association *vs.* no association or positive association *vs.* negative association (Figure 2).

### 3.4. PON1 Genetic Polymorphisms and Enzymatic Activity in CAD

There were seven articles evaluating the influence of Q192R and M55L PON1 polymorphisms on the paraoxonase, arylesterase or hcy-thiolactonase PON1 activity in patients with CAD. There is a complete agreement that the 192R allele increases and the 192Q allele decreases paraoxonase activity in CAD patients, as well as in the healthy population [13,16,17,20]. Similar results have been found for hcy-thiolactonase activity [5,18]. With respect to the M55L polymorphism, the 55L allele has been associated with increased paraoxonase and hcy-thiolactonase activities, while the 55M allele is associated with reduced enzyme activities [16,20,26].

Few data refer to arylesterase activity and its relationship between PON1 polymorphisms in patients with CAD. Jarvik *et al.* [25] reported that the PON1-162/-108/55/192 haplotype did not predict arylesterase activity. However, Liu *et al.* [13] noted that the 192R allele decreases this activity, albeit to a lesser extent to that of paraoxonase activity.

### 3.5. PON1 Enzymatic Activity, Atherosclerosis and CAD

The PON family appears to play an important role in the process of the atheroma plaque formation, but mechanisms are difficult to establish. Several articles support the hypothesis that lower serum PON1 activity is related to an increase in plaque formation and, consequently, to a higher risk of cardiovascular disease. Bayrak *et al.* [17] measured the paraoxonase and hcy-thiolactonase activities of PON1 and the Q192R polymorphism in patients with angiography-detected CAD and in control subjects. Their results showed significantly lower paraoxonase and hcy-thiolactonase activities in the CAD group, together with a non-significant trend towards decreased HDL concentrations. However, Domagala *et al.* [26] observed higher hcy-thiolactonase activities in CAD patients compared to controls; albeit the serum PON1 protein concentration was lower in the CAD group. None of these articles had the measured results adjusted for known risk factors for atherosclerosis (including smoking habit, BMI, serum cholesterol, triglycerides). Conversely, Ferré *et al.* [37] performed a multivariate analysis where the patients were matched for genotype, BMI and age. They found significant differences in paraoxonase and arylesterase activities between patients and controls, but these differences ceased when the genotypes were included in the statistical analysis.

Kabarolgu *et al.* [29] showed that having a low serum PON1 activity was associated with a higher degree of atherosclerosis in patients with confirmed MI or unstable angina pectoris. In addition, several articles [14,22,31] support the hypothesis that lower PON1 activity is correlated with an increase in oxidative stress, leading to the development of atherosclerosis. All of these studies showed significant associations between lower PON1 activity, oxidative stress and CAD. There is no agreement as to whether serum cholesterol and triglyceride concentrations are associated with serum PON1 activity since, despite there being one report [31] of a positive correlation; other reports [14,22] observed a negative correlation. In contrast to these data, investigations conducted in Iran [36] showed no differences in PON1 paraoxonase and arylesterase activities between CAD patients and healthy individuals. Several articles reported lower serum paraoxonase activity in patients with MI in comparison to the control group [18,37,47]. However, it is not known whether this low paraoxonase activity plays a causative role in the pathogenesis of MI or is a consequence of this derangement.

In terms of prevention and prediction of CAD and mortality, a significant correlation was found [16] between lower paraoxonase and arylesterase and an increased risk of major adverse cardiac events (death, myocardial infarction and stroke). Further, these changes in PON activity were related to a greater risk for subclinical myocardial necrosis. Furthermore, lower PON activity was observed in men than in women.

We found one study investigating serum PON1 activity in relation to alterations in coronary blood flow [23]. The results showed that reduced serum PON1 activity might be a biochemical marker of slow coronary flow in patients with angiography-confirmed non-stenosis coronary arteries

In summary, most articles we had identified agreed that serum PON1 paraoxonase activity is decreased in patients with CAD or MI, relative to healthy individuals. Only two articles [20,36] did not find any differences between such groups. Arylesterase activity was reported to be decreased in CAD patients in four articles [13,16,22,27] and unchanged in two [34,36]. More conflict exists regarding hcy-thiolactonase activity. Only two articles studied this parameter, one of which [17] concluded that hcy-thiolactonase activity is decreased in CAD patients, while the other [26] stated that this activity is increased.

## 4. Discussion

Studies of PON enzymes require complex methodology, and small methodological differences can yield contradictory results. For example, with respect to PON1 activity (the most extensively investigated enzyme from the PON family), the current use of synthetic substrates limits the interpretation of the data, since it is not clear whether these measured activities reflect the real endogenous physiological activity of the enzyme. Experimental evidence suggests that, essentially, the lactonase assay measures PON1 that is tightly bound to HDL particles, while the esterase assay measures the tightly-, as well as the loosely-, bound enzyme [48]. The differences between methods, therefore, can be related to differences in the structure and composition of the HDL particles caused by the disease process. There is the possibility that these changes affect PON1 activity, and this is further influenced by the type of substrate used to measure PON1 [49]. Indeed, there is evidence that various elements within PON1’s milieu that regulate its different activities may interact with components of the human carotid atherosclerotic lesions, which are in constant contact with circulating HDL-associated PON1 [50].

Further confusions arise from serum PON1 activity being strongly determined by the enzyme genotype [51,52]. Hence, in case-control studies evaluating enzyme activity, it is imperative that cases and controls are matched for genotype. If not, it would not be possible to ascertain whether the observed changes are due to the disease *per se* or to coincidental differences in allelic frequencies between cases and controls. This is especially true when the reported sample sizes are small, *i.e.*, gene-association studies should be performed with a large sample size involving at least several hundred participants. The extent of the genetic contribution to complex diseases is difficult to ascertain, and the individual contribution of each gene is likely to be modest. Thus, taking into account that the analysis of allele frequencies uses a simple χ^2^ test, the observed associations between a given polymorphism and a particular disease may be merely coincidental. Since a *p*-value of 0.05 is accepted as confirmation of statistical significance, by definition, there is a 5% possibility that a positive result is obtained by chance alone. Increasing the sample size minimizes this risk [53].

Of note is that we found a higher rate of association between PON1 gene polymorphisms and CAD in the articles from 2004 onward, compared to the earlier studies. Of the articles published in the last 10 years, 64% showed an association between PON1 gene polymorphisms and CAD, in contrast to 42% of the articles published before 2004. This observation may be related, perhaps, to changes in the methodology and/or design, and changes in these results may be expected in future investigations.

There is a lack of consensus on the definitions of the outcomes studied, including oxidative stress, arteriosclerosis, artery wall thickness and CAD. Some articles used, as inclusion criteria, the risk factors for arteriosclerosis and previous atherothrombotic events [15], while other studies measured specifically the extent of arteriosclerosis and of CAD diagnosed either with angiography [16], or with electrocardiogram and clinical symptomatology [14], or by measuring coronary blood flow [23]. It is of note that all articles that had studied patients with MI merely considered the surviving subjects in the analyses. This constitutes an important selection bias. Further, comparisons of groups that had included smokers, high-alcohol consumption, hypertension and diabetes mellitus [13,15,18,19,20,21,22,23,24,27,28,29,32,34,39] may need to be re-assessed.

We could find no conclusive relationship between PON1 polymorphisms and CAD or MI. However, there is a consensus on polymorphisms influencing PON1 enzymatic activity, both in the healthy population and in CAD patients. Further, most studies support the concept that alterations in PON1 enzymatic activity levels are associated with oxidative stress, a higher degree of atheromatous plaque formation and poorer clinical outcomes. Nevertheless, there appears to be a step missing that precludes us relating the PON1 genetic polymorphisms with outcomes (CAD or MI). Indeed, variable expressions of PON1, PON2 and PON3 have been detected in macrophages and smooth muscle cells of human atheroma plaques [54,55]. However, the relationships between alterations in the peripheral blood and the expression of the three PON enzymes in human atheroma tissue have not been extensively investigated and are poorly understood [56]. Another aspect of note is the paucity of information on the relationships between PON2 and PON3 gene polymorphisms and enzymatic activities *vs.* cardiovascular disease. This is relevant, since experimental studies in animals and in cultured cells suggest that these two enzymes play an important role in the protection against oxidative stress [5,57,58,59,60]. However, there has been a dearth of physiological evidence in clinical studies relating these enzymes with disease, *i.e.*, their contributions to atherosclerosis and CAD need elucidation. Further, PON1, PON2 and PON3 genes are in genetic disequilibrium. This may imply that the three enzymes influence each other in complex, but as yet unknown, ways [52]. Alterations in the composition and/or structure of HDL particles in the course of development of CAD and MI have been little studied despite the known influence on PON1 activity, irrespective of the genotype [50].

## 5. Conclusions

This systematic review has sought to highlight the gaps in our knowledge of the role(s) that PON enzymes play in the pathogenesis of CAD. We propose the implementation of international, multicenter, multiethnic studies with larger sample sizes to analyze PON1, PON2 and PON3 genetic polymorphisms, the circulating enzyme concentrations and activities and relating these findings in the peripheral blood to changes occurring in tissue during the atherosclerosis process.
